# A Review on Enhancing *Cupriavidus necator* Fermentation for Poly(3-hydroxybutyrate) (PHB) Production From Low-Cost Carbon Sources

**DOI:** 10.3389/fbioe.2022.946085

**Published:** 2022-07-19

**Authors:** Le Zhang, Zicheng Jiang, To-Hung Tsui, Kai-Chee Loh, Yanjun Dai, Yen Wah Tong

**Affiliations:** ^1^ NUS Environmental Research Institute, National University of Singapore, Singapore, Singapore; ^2^ Energy and Environmental Sustainability for Megacities (E2S2) Phase II, Campus for Research Excellence and Technological Enterprise (CREATE), Singapore, Singapore; ^3^ Department of Chemical and Biomolecular Engineering, National University of Singapore, Singapore, Singapore; ^4^ School of Mechanical Engineering, Shanghai Jiao Tong University, Shanghai, China

**Keywords:** polyhydroxyalkanoates, biodegradable plastic, microbial fermentation, waste management, resource recovery, process engineering, metabolic engineering

## Abstract

In the context of a circular economy, bioplastic production using biodegradable materials such as poly(3-hydroxybutyrate) (PHB) has been proposed as a promising solution to fundamentally solve the disposal issue of plastic waste. PHB production techniques through fermentation of PHB-accumulating microbes such as *Cupriavidus necator* have been revolutionized over the past several years with the development of new strategies such as metabolic engineering. This review comprehensively summarizes the latest PHB production technologies *via Cupriavidus necator* fermentation. The mechanism of the biosynthesis pathway for PHB production was first assessed. PHB production efficiencies of common carbon sources, including food waste, lignocellulosic materials, glycerol, and carbon dioxide, were then summarized and critically analyzed. The key findings in enhancing strategies for PHB production in recent years, including pre-treatment methods, nutrient limitations, feeding optimization strategies, and metabolism engineering strategies, were summarized. Furthermore, technical challenges and future prospects of strategies for enhanced production efficiencies of PHB were also highlighted. Based on the overview of the current enhancing technologies, more pilot-scale and larger-scale tests are essential for future implementation of enhancing strategies in full-scale biogas plants. Critical analyses of various enhancing strategies would facilitate the establishment of more sustainable microbial fermentation systems for better waste management and greater efficiency of PHB production.

## Introduction

In the past few decades, the rapid economic development and ever-increasing human living standards greatly increased the demand for plastic products (Narancic et al., 2020). These conventional plastic products are largely made up of petroleum products due to their inexpensive production cost and well-optimized production process. Nevertheless, as the production of petroleum-derived plastics accounted for 12% of global oil consumption, conventional plastic production encountered serious challenges such as non-renewable resource depletion and heightened fuel prices (McAdam et al., 2020). Furthermore, due to the long natural degradation time of petroleum-derived plastics (i.e., decades to hundreds of years) (Wei and Zimmermann, 2017), the extensive use of petroleum-derived plastic products has led to serious environmental issues including white pollution and microplastic problem in both terrestrial and aquatic environment (Xanthos and Walker, 2017; Chae and An, 2018). Hence, there is an urgent need to find an appropriate alternative to petroleum-derived plastics in achieving sustainable plastic production and utilization as well as an environmental-friendly lifecycle.

A potential substituent for conventional plastics was proposed as biodegradable plastics in recent years. Biodegradable plastics essentially refer to biopolymers that are produced by living microorganisms and contain specific monomeric molecules that are covalently bonded by various chemical chains (Aksakal et al., 2021). Since biopolymers such as polysaccharides are commonly produced as storage materials in living cells (Gonzalez-Gutierrez et al., 2010), the biopolymers can be easily depolymerized by certain species of microorganisms such as fungi, yeasts, and bacteria (Sirohi et al., 2020). Hitherto, several kinds of biodegradable plastics produced by microbial cells, including polyhydroxyalkanoates (PHA) (Alves et al., 2022), poly(butylene adipate-co-terephthalate) (Jian et al., 2020), and polylactic acid (PLA) (Taib et al., 2022), have attracted extensive attention worldwide. Among all types of biopolymers, poly(3-hydroxybutyrate) (PHB) has attracted tremendous research attention in recent years due to its high biodegradability (Getachew and Woldesenbet, 2016) as well as significantly lower energy requirement and CO_2_ emission during the production process (Lopez-Arenas et al., 2017). PHB, as one of the short-chain monomers in the polyester families of PHA, has considerably higher crystallinity, better thermal stability, and lower oxygen permeability than other members. Moreover, these physical properties are very similar to those of commercial plastic raw materials such as polypropylene (Markl, 2018), which makes PHB can have a wide range of commercial application scenarios including use as packaging material, coating material in agriculture systems, and carrier material for drug delivery (Boey et al., 2021). Based on this, there is an increasing demand for PHB production globally. As shown by the Nova Institute statistics, the global PHB production was predicted to rise from 34 thousand metric tons in 2013 to around 7.4 thousand metric tons in 2018 (Aeschelmann and Carus, 2015). Nevertheless, the production cost has been a serious issue that hinders the large-scale production of PHB. The PHB production cost was estimated to be 8 to 10 times higher compared to its petroleum-based counterparts (Wang et al., 2013). Specifically, the PHB price ranges from 1.5 to 5.0 € per kg (Chanprateep, 2010), while polypropylene price only ranges from 0.25 to 0.5 US$ per kg (Khanna and Srivastava, 2005). The high production costs led to the fact that the commercial production of PHB had to be limited to small-scale plants with yields ranging from 1,000 to 20,000 metric tons per year (Kosior et al., 2006; Koller and Mukherjee, 2022). Of the total production cost of PHB, the cost of raw materials (carbon sources) constitutes approximately 50–60% (Nonato et al., 2001). In recent years, a burst of research activities has therefore been conducted to synthesize PHB by using more cost-effective carbon sources.

In the application approach of cost-effective carbon sources, microbial fermentation has been considered the most efficient technology for PHB production. Physiologically, PHB is produced as an energy storage and carbon storage material in the cytoplasm of microorganisms under unbalanced nutrient conditions (Tan et al., 2014). Moreover, PHB plays an essential role in enhancing the stress robustness of PHB-accumulating microbial cells (Obruca et al., 2021). Reportedly, there are more than 300 microorganisms that can be used as PHB producers (Grigore et al., 2019), including *Azotobacter, Bacillus, Alcaligenes, Pseudomonas, Rhizobium,* and *Rhodospirillum* (Ertan et al., 2021). However, only a small fraction of bacterial strains have been successfully transferred from laboratory to industry (Możejko-Ciesielska and Kiewisz, 2016). Since *Cupriavidus necator* is one of the most extensively studied bacterial strains for PHB production, it became the first strain being commercially used for PHB production by Imperial Chemical Industries (Verlinden et al., 2007). Many studies have indicated that *C. necator* is able to accumulate a large amount of PHB from various carbon sources. Nevertheless, to the best of our knowledge, currently available reviews related to the production of PHB from *C. necator* are very limited.

Therefore, this mini-review aimed to provide a detailed mechanistic study of the biosynthesis pathway, and a critical overview of the latest research findings regarding PHB production using *Cupriavidus necator* fermentation with different carbon sources. In addition to the conventional process conditions, additional treatment methods and engineering approaches that assisted the bacterial cell biomass and PHB production will also be reviewed. Moreover, this mini-review will also discuss the current challenges and provide future perspectives for large-scale PHB production using *C. necator* strain.

## Mechanisms of Biosynthesis Pathway for PHB Production

**FIGURE 1:**
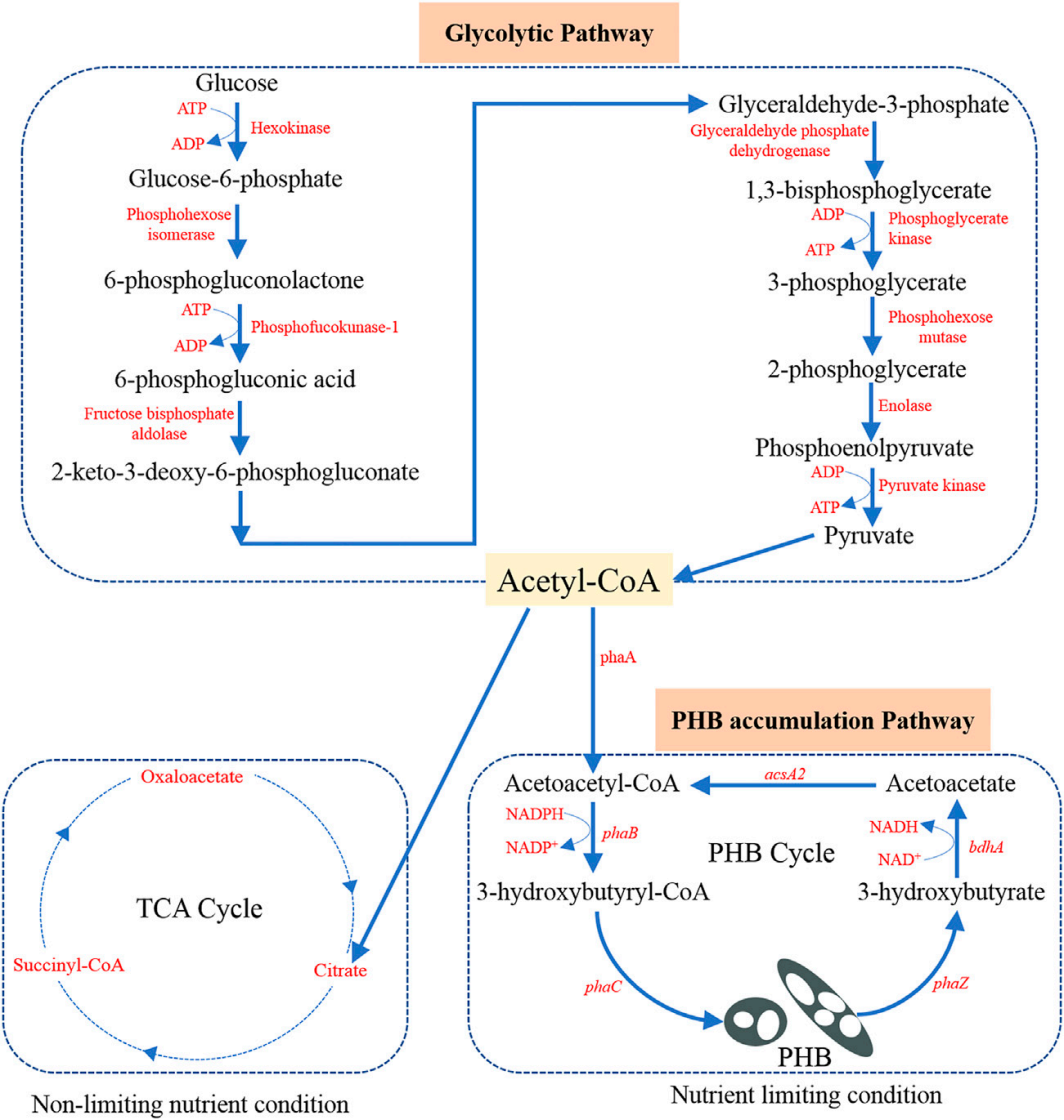
Potential metabolic pathway in *C. necator* from glucose to acetyl-CoA precursor and further to PHB. Adaption from Karthikeyan et al. (2015).

*Cupriavidus necator* (also known as *Ralstonia eutropha*, *Alcaligenes* eutrophus, *Hydrogenomonas eutropha*, and *Wautersia eutropha* in literature) is usually isolated from soil and freshwater. As a result, *C. necator* is well adapted to continuous changes in the environment and allows it to make use of a wide range of organic acids, alcohols, and polyols for its chemoheterotrophic growth (Bowien and Schlegel, 1981; Reinecke and Steinbüchel, 2009). With its excellent metabolic versatility, *C. necator* has become one of the most commonly used microorganisms for PHB production. Although a variety of substrates have been used for PHB production, glucose (usually obtained by hydrolysis of complex molecules) remains the predominant carbon source. As shown in Figure 1, glucose first follows the glycolysis process to produce acetyl-CoA as the precursor for PHB production (Shi and Tu, 2015). The possible glycolysis pathway is called Embden-Meyerhof-Parnas (EMP) glycolytic pathway in *C. necator* (Karthikeyan et al., 2015).

The principal enzymes and the main function genes contained in these enzymes for PHB production are the same in different microorganisms (Reddy et al., 2003). The biosynthesis of PHB involves three main steps and three enzymes (3-ketothiolase, acetoacetyl-CoA reductase, and PHB synthase encoded by corresponding genes named as *phaA*, *phaB,* and *phaC*, respectively), play an important role in the conversion of the acetyl-CoA precursors to PHB (*see* Figure 1). Notably, these three genes *phaA*, *phaB*, and *phaC* are encoded on one operon (phaC, A, and B). Different carbon sources are first converted to the precursor (acetyl-CoA), which is then integrated into the PHB synthesis-depolymerization cycle (Figure 1). The PHB synthesis begins with the condensation of two acetyl-CoA molecules with the help of *phaA* as the catalyst to form acetoacetyl-CoA (Peoples and Sinskey, 1989a). Then acetoacetyl-CoA is reduced (pseudofermentation) to generate 3-hydroxybutyryl-CoA (3HB-CoA) with the help of NADH-dependent *phaB* catalyst (Peoples and Sinskey, 1989b). The monomer 3HB-CoA undergoes polymerization by *phaC* catalyst to produce PHB product in microorganisms (Schubert et al., 1988). It was also found that the constituent monomers are short-chain monomers with 3–5 carbon in *C. necator* while those are medium-chain monomers with 6–14 carbon in *Pseudomonas putida* (Brandl et al., 1988).

In many studies, it has been found that limiting non-carbon nutrients such as nitrogen and phosphate enable PHB accumulation and leads to a higher PHB content per unit of cell dry mass (Schlegel et al., 1961; Wang and Lee, 1997; Jung and Lee, 2000; Ciesielski et al., 2010; Shahid et al., 2013). The underlying metabolic reason is that a shortage of nitrogen supply promotes the regeneration of NADPH (Haywood et al., 1988). NADPH is essential for the *phaB* catalyst to have an effect on the 3HB-CoA formation. Under balanced nutrition conditions, the entrance of the acetyl-CoA into the tricarboxylic acid (TCA) cycle is favored. During the entrance into the TCA cycle, free CoA is released, which can inhibit the action of *phaA* catalyst at high concentrations (Doi et al., 1988). Under unbalanced conditions, the production of free CoA molecules is inhibited and then the metabolism pathway favors the PHB production. Thus, the commonly adopted method to favor PHB production is nitrogen limiting condition which means excess carbon source and limited nitrogen supply.

After the synthesis of PHB in the microorganisms, intracellular degradation can happen if PHB is not recovered in time. Based on Doi et al. (1990), the synthesis and depolymerization of PHB form a cyclic relation (PHB cycle shown in Figure 1). The enzyme PHA depolymerase (*phaZ*) helps to break the polymer bond in PHB and results in the formation of 3-hydroxybutyrate. Then the NADH-dependent dehydrogenase (encoded by gene *bdhA*) enables the oxidation of 3-hydroxybutyrate to form acetoacetate (Delafield et al., 1965). Finally, a synthetase (encoded by gene *acsA2*) allows the regeneration of acetoacetyl-CoA to be used for PHB synthesis again (Cai et al., 2000). With an improved understanding of the aforementioned biochemical and metabolic mechanisms, it would be possible to efficiently regulate the PHB biosynthesis and maximize the PHB accumulation in *C. necator*.

## Current Carbon Sources and PHB Production Efficiencies

### PHB Production From Food Waste

Biodegradable PHB has attracted great research attention as an alternative to commercial petroleum-based plastics for the sustainable target. However, the high production cost has undermined the economic viability of PHB commercial production. Many studies have been done to find cheap and renewable carbon sources for PHB production such as edible oil and raw starchy food product (Kek et al., 2010; da Cruz Pradella et al., 2012; Ali and Jamil, 2014; Priji et al., 2016). Nevertheless, the ever-growing food demand has intensified the competition with our food supply and thereby threatened food security. Therefore, utilizing food waste that has the same chemical composition as the unused food products could be a promising and sustainable carbon source for PHB production.

In recent years, food waste has become a serious problem to global food security and sustainable development goals stated by the United Nations (Otles et al., 2015; Lee et al., 2016). In addition, the loss of food also leads to the loss of other resources such as energy and manpower used in the supply chain. Around one-third of the food produced have been wasted annually (Gustavsson, 2011). Utilizing food waste for the production of the value-added PHB could effectively lower the overall production cost and at the same time curb the environmental issues arising from the ever-growing food waste problems. For instance, chicory roots as an abundant food residue in Spain were first hydrolyzed and then used as fermentation substrate for three PHB-producing strains, i.e., *C. necator* DSM 428, DSM 531, and DSM 545 (Haas et al., 2015). The results showed that a dry biomass concentration of 3.5–14.0 g/L and a PHB content of 46–78% in dry mass were achieved. Volatile fatty acids derived from dairy waste were successfully utilized as a carbon source for PHB accumulation in strain *C. necator* DSM 13513 (Pagliano et al., 2020). A summary of studies on food wastes used as carbon sources and corresponding PHB production yields is presented in Table 1.

**TABLE 1:** Summary of PHB Production using *C. necator* from different carbon sources.

Carbon source	Strain	Dry cell weight (DCW) (g/L)	PHB content (%)	PHB yield (g/g)	PHB productivity (g/(L·h))	Reference
Waste frying rapeseed oil	*C. necator* H16	2.95	41	—	—	Verlinden et al. (2011)
Waste sesame oil	*C. necator* H16	7.9	62.3	—	—	Obruca et al. (2013)
Used cooking oil	*C. necator* DSM 428	11.6	63	0.77	0.15	Cruz et al. (2015)
Waste frying oil	*C. necator* H16	4.2	79.2	—	—	Riedel et al. (2015)
Waste animal fats	*C. necator* H16	2.5	61.3	—	—	Riedel et al. (2015)
Sugarcane molasses	*C. necator* DSM 545	17.07	44	0.22	0.12	Beaulieu et al. (1995)
Sugarcane molasses	*C. necator*	2.86	27	—	—	Sen et al. (2019)
Sugarcane vinasse and molasses	*C. necator* DSM 545	20.9	56	—	—	Dalsasso et al. (2019)
Beet molasses	*C. necator* ATCC 25207	28.89	52.89	0.5	0.33	Ertan et al. (2021)
Waste potato starch	*C. necator* NCIMB 11599	179	52.5	0.22	1.47	Haas et al. (2008)
Broken rice	*C. necator* DSM 545	13.32	44.09	—	0.054	Brojanigo et al. (2020)
Purple sweet potato	*C. necator* DSM 545	10.48	34.42	—	0.038	Brojanigo et al. (2020)
Paddy straw	*C. necator* MTCC 1472	19.2	27.03	—	—	Sandhya et al. (2013)
Wheat straw	*C. necator* ATCC 17699	15.3	65	0.16	—	Dahman and Ugwu, (2014)
Wheat bran	*C. necator* NCIMB 11599	24.5	62.5	0.319	0.255	Annamalai and Sivakumar, (2016)
Kenaf biomass	*C. necator* ATCC 17699	19.35	57.6	0.454	—	Saratale et al. (2019)
Wheat straw	*C. necator* DSM 545	15.1	80.1	0.017	0.252	Soto et al. (2019)
Hemp hurd biomass	*C. necator* ATCC 17699	23.8	56.3	0.253	0.139	Khattab and Dahman, (2019)
Waste glycol	*C. necator* DSM 545	68.8	38	0.34	0.84	Cavalheiro et al. (2009)
Gas mixture (H_2_:CO_2_:O_2_=75:10:15)	*C. necator* ATCC 17697	60	60	—	0.6	Ishizaki and Tanaka, (1991)
Gas mixture (H_2_:CO_2_:O_2_=75:10:15)	*C. necator*	60	82	—	0.41	Mozumder et al. (2015)
Gas mixture (H_2_:CO_2_:O_2_=84:13.2:2.8)	*C. necator* DSM 545	19	61	—	0.168	Garcia-Gonzalez et al. (2015)
Gas mixture (H_2_:CO_2_:O_2_=70:10:20)	*C. necator*	5	67	—	0.052	Lu and Yu, (2017)
Syngas (H_2_:CO_2_:CO:N_2_=20:20:20:40)	*C. necator* H16	33.8	42	0.54	0.189	Shin et al. (2021)
Gas mixture (H_2_:CO_2_:O_2_=7:1:0.25)	*C. necator* H16 (gene modified)	0.55	50.4	—	—	Tang et al. (2020)
Gas mixture (H_2_:CO_2_:O_2_: N_2_=3.6:12.3:7.6:76.5)	*C. necator* H16 (gene modified))	0.39	70	—	0.002	Miyahara et al. (2020)

#### PHB Production From Lipid-Rich Food Waste

Lipid-rich food waste, which includes waste vegetable oil and animal fat has high fatty acid content. This makes them suitable for PHB production as fatty acid is able to deliver more energy as a carbon source compared to the commonly used glucose substrate (Solaiman et al., 2006). Compared to the complete oxidation of glucose during the metabolic process, fatty acid undergoes complete β-oxidation, which is able to generate more ATP and more acetyl-CoA molecules, which result in higher substrate-to-PHB yields (Talan et al., 2020). Large quantities of lipid-rich food waste are generated annually and due to their high chemical and biological oxygen demands, additional treatment needs to be adapted to properly dispose of these wastes (Jiang et al., 2016). Because of the nature of waste vegetable oils, they usually have undergone high-temperature processing such as frying or cooking. This typical feature makes them unsuitable for animal feed due to the toxic compounds formed under high temperatures but allows them to be used directly for PHB production without any pretreatment (Pernicova et al., 2019). Initially, some researchers have studied the production efficiency using single waste vegetable oils for PHB production with *C. necator*. Verlinden et al. (2011) used waste frying rapeseed oil as the single carbon source with *C. necator* and achieved 2.95 g/L biomass content and 41% PHB accumulation. Obruca et al. (2013) used waste sesame oil as the single carbon source with *C. necator* and achieved 7.9 g/L biomass content and 62.3% PHB accumulation. However, without an additional collection or separation process, the waste oils usually exist in a mixture form instead of containing only a single oil type. Cruz et al. (2015) started to study the PHB production using a mixed waste cooking oil and obtain a relatively high biomass accumulation of 11.6 g/L and PHB content of 63%. Unlike vegetable oil, waste animal fat contains high content of saturated fatty acids and usually exists in a solid form under room temperature which makes additional treatment such as emulsification to be mandatory. Such limitation makes using waste animal fat as the carbon source for PHB production to be scarce. Moreover, Riedel et al. (2015) have compared the PHB production yield using waste frying oil and waste animal fat with *C. necator* and found that frying oil tended to produce more PHB (3.33 g/L) compared to animal fat (1.53 g/L). Koller and Braunegg (2015) used animal-based crude glycerol and saturated biodiesel share as the carbon source of *C. necator* strain DSM 545 for PHB production. The obtained PHB showed high versatility of biopolymer properties that made them applicable in various fields of the plastic market.

#### PHB Production From Sugar-Rich Food Waste

Molasses, which is a viscous residue produced during the sugar crystallization process of the sugar production industry, is a widely available waste stream due to the steadily expanding global sugar industry. Due to its high content of sucrose with some glucose and fructose, molasses has been widely used as a cheap carbon source in the fermentation process for biofuels and biopolymers production (Raza et al., 2018). Sugarcane molasses has been utilized as the carbon source for *C. necator* growth and PHB production, which achieved 17.07 g/L biomass content and 44% PHB accumulation (Beaulieu et al., 1995). Later, in Sen et al. (2019), they have explicitly studied the effect of different pretreatment methods on the PHB production with strain *C. necator* and found that hydrothermal acid pretreated molasses resulted in the highest PHB accumulation of 0.78 g/L and PHB content of 27.3% due to more complete hydrolysis. On the basis of this study, recently, Dalsasso et al. (2019) proposed that supplementing molasses with vinasses can promote cell growth and hence result in a better PHB accumulation of 11.7 g/L and PHB content of 56% due to the effect of a complex mixture of nutrients. Most recently, random mutagenesis of the microorganism was adopted to allow the fructose-preferring strain *C. necator* ATCC 25207 to be able to utilize molasses as the nutrient source and achieved a relatively high PHB accumulation of 15.28 g/L and PHB content of 52.89% (Ertan et al., 2021).

#### PHB Production From Starch-Rich Food Waste

Starch is a complex polysaccharide that consists of various glycosidic bonded glucose monomers. As a widely available hydrocarbon in natural plants, starch could be a potential cheap carbon source for microbial growth. However, due to its complex nature, the utilization of starch as a nutrition source is limited and usually requires additional pretreatment such as hydrolysis to break the complex starch into single glucose units. It has been reported that bakery waste, a kind of starch-rich food waste, was successfully used as a carbon source for PHB production through fermentation of PHB-accumulating microbes (Pleissner et al., 2014). Haas et al. (2008) utilized waste potato starch as the carbon source for *C. necator* growth and PHB production, which achieved 179 g/L biomass accumulation and 52.5% PHB content. Later, in Brojanigo et al. (2020), they have explicitly studied the PHB production efficiency using various starchy materials with strain *C. necator* and found that broken rice resulted in the highest PHB accumulation of 5.87 g/L and PHB content of 44.09% due to less residue starch granules after hydrolysis. More recently, Brojanigo et al. (2022) developed a recombinant amylolytic strain of *C. necator* DSM 545 for the one-step PHB production using starchy residues (i.e., broken rice and purple sweet potato waste) as carbon sources. Both the *G1d* gene encoding glucodextranase and the *amyZ* gene encoding α-amylase were expressed in *C. necator* DSM 545. The recombinant *C. necator* DSM 545 produced 5.78 g/L of biomass and 3.65 g/L of PHB, respectively.

### PHB Production From Lignocellulosic Materials

Lignocellulosic material, which consisted of cellulose, hemicellulose, and lignin has been underutilized for the microbial fermentation process due to its recalcitrant nature. Additional pretreatment method is always required to remove the lignin to break down the connection between cellulose and hemicellulose and increase surface area for further microbial fermentation (Sun and Cheng, 2002). Tomizawa et al. (2014) have observed that few microbial strains can utilize lignocellulosic material as the sole carbon source for their growth. Sandhya et al. (2013), attempted to use paddy straw as the single carbon source with *C. necator* and achieved 19.2 g/L biomass accumulation and 27.03% PHB content. Dahman and Ugwu (2014) used wheat straw and achieved 15.3 g/L biomass accumulation and 65% PHB content. Similarly, Annamalai and Sivakumar (2016) took use of wheat bran hydrolysate as the carbon source and obtained a better biomass production of 24.5 g/L and a similar PHB content of 62.5%. Kenaf biomass, which is another widely available lignocellulosic material was utilized by Saratale et al. (2019) for PHB production and a relatively high PHB production yield of 0.454 g per g reducing sugar was achieved. Later, Soto et al. (2019) continued to study the PHB production using a wheat straw with another *C. necator* strain *(C. necator* DSM 545) and achieved a higher PHB accumulation of 12.1 g/L and PHB content of 80.1%.

Hitherto, *C. necator* has been confirmed as a glucose-utilizing strain with high PHB-production ability; however, the conversion of pentoses (e.g., xylose) derived from hydrolysis of the hemicellulose content in lignocellulosic materials has seldom been reported. Recently, Lee et al. (2021) found that the co-culture system of *C. necator* NCIMB 11599 and *Bacillus* sp. SM01 contributed to the enhancement of PHB production and utilization of xylose as a carbon source. More studies can be carried out to improve the conversion of pentose (e.g., xylose) during PHB production *via C. necator* strain. Furthermore, when hydrolysates of the lignocellulosic materials are used as carbon sources for *C. necator* fermentation, the potential growth inhibitors (e.g., acetic acid, furfural, and 5-hydroxymethylfurfural) in the lignocellulosic hydrolysates could potentially inhibit the microbial cell growth and PHB accumulation (Marudkla et al., 2018). Hitherto, the effects of levulinic acid (Novackova et al., 2019), benzoic acid, p-coumaric acid, furfural (Wang et al., 2014), and acetic acid (Marudkla et al., 2018) on cell growth and PHB production have been investigated. It was found that the lignocellulosic hydrolysates after necessary detoxification can be a low-cost alternative substrate of commercial glucose to economically produce PHB (Kucera et al., 2017; Dietrich et al., 2019). Previous studies demonstrated that agro-industrial residues (lignocellulosic materials) can offer cost benefits for PHB production (Manikandan et al., 2020; Sirohi et al., 2020). Hence, with further consideration of by-products and other useful resources generated from the PHB production process from lignocellulosic materials, more values can be achieved.

### PHB Production From Glycerol

Crude glycerol generated during the transesterification process is the main by-product of the biodiesel industry. Although pure glycerol has high commercial value as it can be used as a feedstock for a wide range of industries such as the food and cosmetics industry, crude glycerol is usually regarded as a waste rather than a co-product due to the presence of impurities such as methanol and sodium chloride (Wang et al., 2001). Additional purification steps are required to turn glycerol into a valuable by-product. Due to the increasing production capacity of biodiesel, the large volume of crude glycerol is produced annually and adds burdens to the associated disposal cost. There is a need to find an upgrade to add value to crude glycerol. Since glycerol occurs in nature, many microorganisms can utilize glycerol for their metabolism activities, which makes glycerol a potential carbon source for PHB production. In Cavalheiro et al. (2009), the PHB production using commercial and waste glycerol with *C. necator* was studied. The results showed that the maximum PHB yields were 51.15 and 26.14 g/L, while the PHB contents were 62 and 38%, respectively. Since methanol is the principal impurity in crude glycerol and is generally considered toxic to microbes, Salakkam and Webb (2015) studied the inhibition effect of methanol in PHB production and reported the existence of methanol has a negative effect on cell growth for *C. necator*. Due to the fact that the cell growth rate of *C. necator* in glycerol is 11.4 times lower than in gluconate, González-Villanueva et al. (2019) adopted adaptive laboratory evolution to enhance the glycerol assimilation in *C. necator* H16. The obtained new strain after adaptation showed a 9.5 times higher specific growth rate than the wild-type strain. Moreover, Strittmatter et al. (2022) reported that *C. necator* H16 can grow on glycerol through autotrophy metabolism mode, while it can also grow through mixotrophy metabolism mode with simultaneous glycerol utilization and CO_2_ fixation. More insights into the effects of the fermentation conditions on cell metabolism would contribute to the enhancement of PHB production by *C. necator* through model-guided bioprocessing. In this regard, Sun et al. (2020) used a constrained-based stoichiometric modeling approach to study the metabolic change associated with PHB synthesis during fermentation with glycerol.

### PHB Production From Carbon Dioxide

Carbon dioxide (CO_2_) is the major greenhouse gas produced by human activities that can lead to global warming (Tollefson, 2009; Federsel et al., 2010). The excess emission of CO_2_ has become a serious environmental problem in many countries (Shukla et al., 2010). This leads to an urgent need for the science community to develop an efficient approach to mitigate CO_2_ emissions. Among the different approaches developed, utilizing CO_2_ as the raw material for the production of more valuable chemicals has attracted great research attention (Liu et al., 2015; Li et al., 2018). Among various CO_2_ valorizations, microbial fermentation for PHB production has gained great attention in recent years. *C. necator*, which is a gram-negative facultatively chemoautotrophic bacterium, is able to assimilate CO_2_ to produce PHB through the Calvin–Benson–Bassham (CBB) cycle (Bowien and Kusian, 2002). Unlike other organic carbon sources, which require heterotrophic growth, *C. necator* can utilize CO_2_ through H_2_ oxidation under autotrophic growth conditions (Li et al., 2020). Ishizaki and Tanaka (1991) attempted to utilize a gas mixture (H_2_:CO_2_:O_2_=75:10:15) as the carbon source and achieved 60 g/L biomass accumulation and 60% PHB content. Later, Mozumder et al. (2015) studied the effect of gas mixture composition and developed a corresponding model to find the optimal gas composition for PHB production, where a maximum biomass accumulation of 60 g/L and PHB content of 82% occurred at the gas composition of H_2_:CO_2_:O_2_=75:10:15. Moreover, to ensure the experimental results can be scalable to the industrial scale, Garcia-Gonzalez et al. (2015) purposely kept the O_2_ concentration below the industrial safety margin (H_2_:CO_2_:O_2_=84:13.2:2.8) and obtained a biomass accumulation of 19 g/L and PHB content of 61% using a two-stage cultivation system. Given a gas mixture is used as the carbon source for cell growth, Lu and Yu (2017) proposed to use a packed-bed reactor instead of a stirred tank reactor to improve the mass transfer between cells and feed gas, resulting in a biomass accumulation of 5 g/L and PHB content of 67%. Other than mixing CO_2_ with H_2_ and O_2_ as a carbon source, synthetic gas (syngas), which is a common industry effluent produced from pyrolysis and steam reforming, can be directly used as the carbon source for PHB production (Liu et al., 2014). Shin et al. (2021) utilized syngas (H_2_:CO_2_:CO:N_2_=20:20:20:40) to achieve a biomass accumulation of 33.8 g/L and PHB content of 42%. In recent years, many researchers started to adopt metabolic engineering techniques for more efficient PHB production from CO_2._ Tang et al. (2020) constructed a recombinant strain by gene modification in *C. necator* to achieve a cell accumulation of 0.55 g/L and PHB content of 50.4%. Similarly, Miyahara et al. (2020) inserted a gene encoding PHA synthase 1 from *Pseudomonas* sp. into *C. necator* to better utilize CO_2_. In addition, since H_2_ oxidation is required for cell growth, to avoid explosion during the fermentation process, Miyahara et al. (2020) utilized a gas mixture with a composition of H_2_:CO_2_:O_2_:N_2_= 3.6:12.3:7.6:76.5. With such a low H_2_ concentration, a biomass accumulation of 0.385 g/L and PHB contents of 70% were achieved. Likewise, Lambauer and Kratzer (2022) recently reported a lab-scale cultivation of *C. necator* on an explosive gas mixture of H_2_, CO_2_, and O_2_ in a ratio of 85:10:2. The results showed that the CO_2_ was fixed during the cultivation and that approximately 98% of the produced PHB was formed from CO_2_. Indeed, the gene for CO₂ fixation has been identified in *C. necator*, which allows its heterotrophic growth using CO_2_ as a carbon source (Bellini et al., 2022).

## Current Strategies Used to Enhance PHB Production Efficiencies

### Pre-Treatment Methods

Cheap and renewable carbon sources generally consist of complex compounds such as lipid, starch, and lignocellulose. These materials cannot be directly consumed by *C. necator* to produce PHB through microbial fermentation. Therefore, before the real fermentation step, hydrolysis is usually necessary to convert the raw carbon sources into monomeric glucose for the metabolism processes. Acid and enzymatic hydrolysis have been widely used in the production of PHB with *C. necator*. The choice of hydrolysis methods always depends on the justification of cost and hydrolysis efficiency. Dalsasso et al. (2019) have compared the effect of acid and enzyme hydrolysis on PHB production and found out enzymatic hydrolysis gives a better specific growth rate. They concluded that acid hydrolysis has the advantage of low-cost processing, while enzymatic hydrolysis gives a more complete hydrolysis conversion. To utilize the benefits of both methods, Azizi et al. (2017) chose to use a combined method to produce PHB from brown seaweed using *C. necator*.

In addition, the presence of recalcitrant compounds such as starchy and lignocellulosic carbon sources always limits the hydrolysis conversion (Oh et al., 2015). Additional pretreatment methods are always adapted by researchers to further improve the hydrolysis efficiency. Alkaline pretreatment is one of the commonly used chemical pretreatment methods. It is a cheap, simple, and effective method that helps to increase the contacting surface area for better hydrolysis (Kim et al., 2016). Saratale et al. (2020) have studied the effects of various pretreatment methods on the PHB production using *C. necator* and found out that a mild alkaline pretreated kenaf biomass allows a maximum PHB accumulation of 10.10 g/L and a PHB yield of about 0.488 g/g of reducing sugar. Moreover, alkaline pretreatment liquor (APL) can preserve more than 90% of the sugar (Xu and Cheng, 2011). Due to the high sugar preservation, Li et al. (2019) improved the PHB production efficiency (by 10-fold) by utilizing APL with an oxidative enzyme-mediator-surfactant system using *C. necator.*

On top of the alkaline pretreatment, physical pretreatment methods such as hydrothermal, microwave, and ultrasound are also common methods used in research studies to improve hydrolysis efficiency by lowering the reaction activation energy (Kang et al., 2019). For instance, Ravindran et al. (2018) tested the effect of six pretreatment methods on the extent of hydrolysis of brewers’ spent grain (BSG). They found that microwave-assisted alkali pretreatment allowed the reducing sugar yield to achieve as high as 0.228 g reducing sugar/g BSG. Moreover, ultrasound pretreatment can also increase the hydrolysis extent through a more efficient structural deconstruction of recalcitrant compounds. For example, Deshmukh et al. (2020) have taken the use of ultrasound and successfully improved the PHB production yield by 1.98 fold using *C. necator.* Similarly, Saratale et al. (2020) combined the ultrasound with alkaline pretreatment and achieved a relatively high PHB accumulation of 7.85 g/L and PHB yield of about 0.441 g/g of reducing sugar produced from wheat waste using *C. necator.*

### Fermentation Strategies

#### Nutrient Limitation

*C. necator* is a typical type of non-growth associated PHB-producing bacteria. Therefore, non-carbon nutrients’ limitation has been a widely used approach for *C. necator* to inhibit biomass production and enable intracellular PHB accumulation (Kachrimanidou et al., 2014). In most of the studies, the nutrient limiting conditions, which generally refer to depletion of nitrogen and phosphorus, are complemented to improve the PHB production yield using *C. necator* (Ryu et al., 1999; Nygaard et al., 2019). Among the nutrient limiting conditions, nitrogen limitation is the more commonly used approach (López-Cuellar et al., 2011; Wu et al., 2013). In some of the studies, researchers completely removed the nitrogen source to improve PHB accumulation (Vadlja et al., 2016). Under these extreme cases, pH control is monitored through NaOH instead of NH_4_OH to achieve a 0% nitrogen supply. However, it was also proved by several studies that PHB production in *C. necator* could be negatively affected due to the accumulation of Na^+^ ions when NaOH is used (Yeo et al., 2008; Passanha et al., 2013). In addition to nitrogen limitation, phosphate limiting conditions have a similar effect to stimulate intracellular PHB accumulation (Shang et al., 2007; Grousseau et al., 2013). The trigger mechanism of phosphate limiting conditions can be related to the fact that phosphorus feeding influences the NADPH availability and resultant PHB accumulation kinetics (Grousseau et al., 2013).

Moreover, many studies have also found that although a high C/N ratio or a completely 0% nitrogen supply led to high PHB accumulation in *C. necator*, this extreme condition can also lead to low biomass production which resulted in an overall low PHB content (Kachrimanidou et al., 2014; Bhatia et al., 2018). Yang et al. (2010) have studied the effect of the C/N ratio on PHB production in *C. necator*. They found that a high C/N ratio (∼80) gave an optimal PHB accumulation while a medium C/N ratio of 40 led to maximum biomass production. Chanprateep et al. (2008) also found that a low or medium C/N ratio ranging from 4 to 20 resulted in a maximum PHB production rate in *C. necator*. Similarly, Park and Kim (2011) varied the C/N ratio and found that the optimal C/N ratio for both biomass production and PHB accumulation in *C. necator* was 20. Bhatia et al. (2018) also found that a C/N ratio of 20 resulted in the optimal PHB content of 53.8%.

#### Feeding Optimization Strategies

The feeding strategy, which mainly refers to the way in which carbon sources are fed into the fermentation system, has a great effect on the PHB accumulation in *C. necator*. The feeding strategy includes one pulse feeding, multi-pulse feeding, and continuous feeding. Some researchers pointed out that multiple feeding regimes or fed-batch technology have shown improved PHB production compared to the one-stage batch fermentation process (Mozumder et al., 2014; Garcia-Gonzalez et al., 2015; Keunun et al., 2018). The underlying reason is still the nutrient limitation required by PHB accumulation in *C. necator*. The multi-pulse feeding or multi-stage fermentation allows the cell growth and PHB accumulation to take place in separate steps. Hence, in the first stage, the *C. necator* is allowed to grow into high cell density under nutrient-balanced conditions until enough biomass is produced. Then, in the second stage, the nutrient limiting condition is applied so that PHB accumulation starts to speed up, while biomass production is restricted. In contrast, in a single-stage fermentation process, nutrient-balanced conditions will result in fast cell growth but limited PHB accumulation in *C. necator*; while the nutrient limitation condition will result in high PHB accumulation but limited biomass produced. Both conditions in the single-stage fermentation process give a lower final PHB content compared to the multi-stage fermentation process. Atlić et al. (2011) found that continuous production of PHB by *C. necator* in a multi-stage bioreactor showed high volumetric productivity of 1.85 g/(L·h) and high PHB content of 77% (w/w). Hafuka et al. (2011) investigated the effects of feeding regimes on PHB production in *C. necator* in detail and concluded that one pulse feeding resulted in the highest biomass production, while multi-steps and continuous feeding gave a continuously high PHB production. Another good example of continuous feeding was shown in Kedia et al.’s study, which demonstrated that a more continuous feeding regime by using an automatic feed method on basis of the pH control increased the conversion rate of acetic acid and butyric acid to PHB by approximately 2-fold (Kedia et al., 2014). Similarly, Mozumder et al. (2014) developed a three-stage feeding strategy to control the carbon source concentration for PHB production in *C. necator* and achieved a maximum biomass accumulation of 104.7 g/L and PHB content of 65.6%. Furthermore, other innovative regimes such as cell recycling approaches in a membrane bioreactor also showed high PHB productivity (i.e., 3.1 g/(L·h)) and good yield (i.e., 0.33 g PHB/g added glucose) (Haas et al., 2017).

### Metabolic Engineering Strategies

*C. necator* is the most promising and well-studied bacterium for PHB production. Due to its potential for large-scale PHB production, many researchers focus on improving production efficiency using *C. necator*. Other than pretreatment methods and fermentation strategies, more and more researchers focus on metabolic engineering on *C. necator*. Indeed, to facilitate PHB accumulation, the native metabolic pathways in the wild-type of *C. necator* need to be modified using metabolic engineering strategies, systems, and synthetic biology approaches. The main purposes of genetic modification usually include increasing PHB content and broadening the choice of carbon sources for *C. necator*. Sucrose has attracted much research attention as a potential substrate for PHB production due to its low cost and large supply (Sabri et al., 2013). However, most of the wild types of *C. necator* are unable to assimilate sucrose. Park et al. (2015) made sucrose utilization in *C. necator* NCIMB11599 possible by inserting the *sacC* gene that encodes β-fructofuranosidase from *Mannheimia succiniciproducens* MBEL55E, achieving about 1.96 g/L PHB titer and 73.2 wt% PHB content. The introduction of the highly active β-fructofuranosidase allows fast conversion of sucrose into glucose. Different from *sac* gene insertion case in which *C. necator* can utilize glucose and fructose obtained from sucrose hydrolysis, Arikawa et al. (2017) inserted the sucrose utilization *csc* genes from *Escherichia coli* W into *C. necator* H16 to make direct uptake of sucrose possible and improved the PHB production by 1.5-fold compared to using glucose as sole carbon source.

Moreover, acetyl-CoA is one of the key intermediates for the PHB biosynthesis in *C. necator*. Lee et al. (2003) transformed *tktA* gene, which boosts the formation of acetyl-CoA and encodes transketolase from *E coli* into *C. necator* H16. The authors successfully improved the PHB production by around 1.6-fold compared to the non-modified *C. necator* H16. Furthermore, oxygen limiting condition is a common condition for PHB production in *C. necator* using CO_2_ due to safety considerations and PHB accumulation requirements. However, most genes related to energy generation and key intermediates formation are downregulated under oxygen-limiting conditions. Tang et al. (2020) genetically engineered *C. necator* H16 through heterologous expression of *vgb* and *ldh* and increased both the biomass and PHB production by around 30%. Furthermore, two genes involved in PHB metabolism, *phaA,* and *phaB*, from the strain *C. necator*, were utilized for the creation of a new metabolic engineering strategy based on the regulatory protein PirC (Koch et al., 2020). Špoljarić et al. (2013) established two mathematical models for fed-batch production of PHB by *C. necator* DSM 545 on glycerol, fatty acid methyl esters as well as glucose and valeric acid. These two mathematical models were successfully used to optimize the feeding strategies of mixed carbon and nitrogen sources for PHB production by *C. necator* on combined substrates. In another computational optimization study by Horvat et al. (2013), PHB productivity was enhanced from experimentally 2.14 g/(L·h) to simulated 9.95 g/(L·h) by the applied Luedeking-Piret’s model. Recently, Panich et al. (2021) reviewed the technological advances in metabolic engineering of the hydrogen-oxidizing bacterium *C. necator* H16. On the one hand, metabolic engineering approaches can be utilized for enhancing the biosynthesis of PHB by altering the PHB biosynthesis pathways. On the other, by using metabolic engineering techniques, carbon flux can be directed away from PHB synthesis toward the generation of biofuels and bioproducts such as isoprenoids, terpenes, isobutanol, alkanes, and D-mannitol (Hanko et al., 2022).

## Challenges and Future Prospects

**FIGURE 2:**
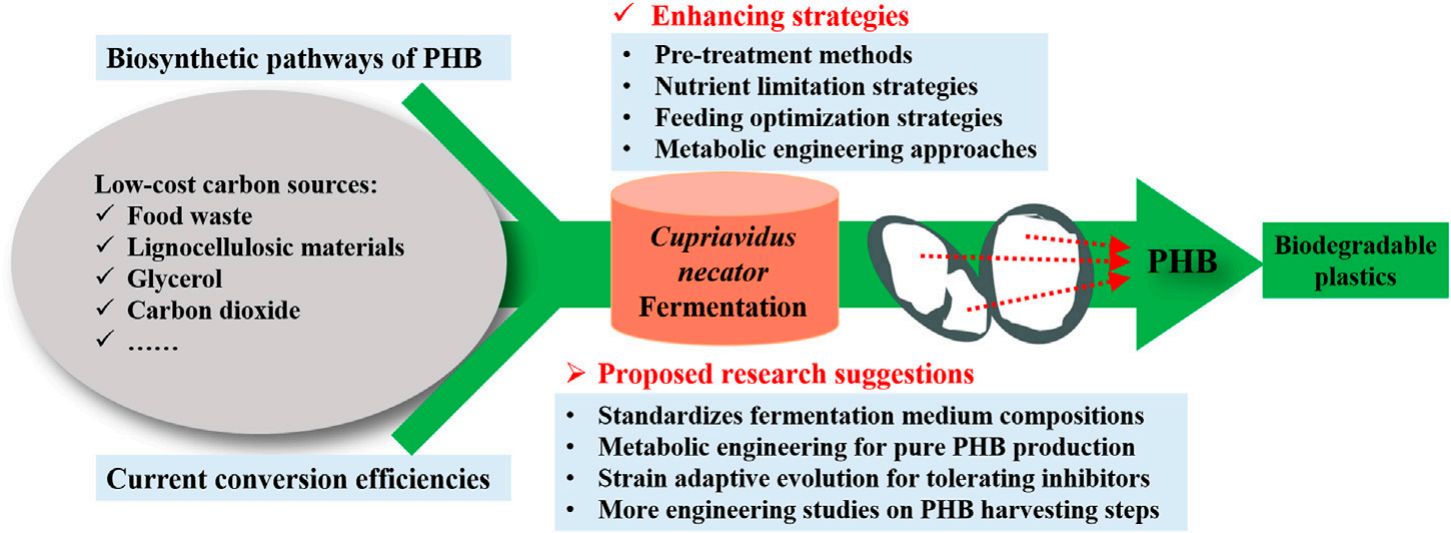
Proposed suggestions for further increase of the PHB yield and productivity from *C. necator* fermentation.

However, there are still several challenges that need to be overcome before the scale-up application of the PHB production. Currently, the production cost is still the greatest challenge hindering the expansion of the commercial PHB market. Even using cheap biodiesel industry by-products, the economic evaluation by Kachrimanidou et al. (2021) still pointed out that the large-scale PHB production was not sufficiently cost-effective compared to its petroleum-derived counterparts, due to its much higher production cost. However, life cycle assessment confirmed that PHB production is much more sustainable compared to conventional plastic due to its much lower greenhouse gas emissions, non-renewable energy use, and abiotic depletion potential (Kookos et al., 2019). Therefore, the future study of PHB production should focus on producing cost-effective PHB from low-cost substrates and a well-optimized production process. In order to further increase the PHB yield and productivity of *C. necator*, several suggestions are proposed (Figure 2) for future studies.

The properties of PHB produced largely depend on the type and composition of carbon sources used. Since more and more researchers focus on renewable sources such as organic waste streams to reduce the production cost, the compositions of the waste streams are likely to vary, which may make the quality of the PHB produced to be inconsistent. Therefore, additional controlling approaches such as nutrient supplementation are required to standardize the fermentation medium composition. More studies should be done to determine the effect of medium composition on the characterization of PHB produced. Additionally, it can be a promising way to achieve commercialization of PHB production from *C. necator* through highly efficient utilization of lignocellulosic hydrolysate as the nutrient source. Nevertheless, the fermentation inhibitors generated in the hydrolysis process of lignocellulosic biomass could hinder microbial cell growth and PHB accumulation. To solve this issue, more research can be done to develop novel *C. necator* strains with the ability to tolerate the typical inhibitors (i.e., furfural, 5-hydroxymethylfurfural, and acetic acid) in the hydrolysate of lignocellulosic materials. Notably, strain adaptive evolution for tolerating inhibitors coupled with selection can be a promising approach. Moreover, genetic manipulation of *C. necator* has been widely studied. Currently, it focuses on improving PHB production efficiency and broadening the utilizable substrate range. However, the production of by-products is another common problem in PHB production. The existence of by-production affects the PHB production yield and adds an extra burden to the downstream purification process. Therefore, the future works of metabolic engineering should focus on the production of a single product to improve the efficiency of carbon source utilization.

Furthermore, few studies have been done to study the engineering aspect of PHB production. The engineering aspect of PHB production steps such as pretreatment, reactor, extraction, and purification parameters have been the key factor to be considered during the scale-up application. This suggests there is still a blank in the reduction of processing cost by optimizing both the upstream and downstream processes. Future studies should focus on all processing stages and PHB production at pilot-scale and pre-commercial scales should be conducted to further optimize the processing cost.

## Conclusion

*C. necator* has been one of the most promising microbial strains used for PHB production. This review has shown that the PHB production using cheap and renewable carbon sources have a great potential to be scaled up for commercial production. This study provided a summary of various carbon sources and common strategies that the researchers adopted to improve PHB production. Currently, the procurement cost and renewability of raw materials are still the main challenges hindering the production of biodegradable PHB plastic on an industrial scale. To enhance overall PHB yield, hydrolysis is the commonly adopted method to convert the complex organic carbon sources into utilizable glucose for PHB production in *C. necator.* In this regard, enzymatic hydrolysis can give a more complete conversion of organic waste to glucose, compared to acid hydrolysis; however, the high operation cost undermined the economic viability of this approach. Meanwhile, physical and chemical pretreatments are usually used as a complement to improve hydrolysis efficiency. To promote PHB accumulation in *C. necator*, a careful selection of the carbon to nutrients (i.e., nitrogen and phosphorus) ratio is necessary to ensure the balanced nutrients condition. Regarding the system design aspect, multi-stage fermentation has the advantage of both sustainable microbial growth and high PHB accumulation by separating the cell growth and PHB accumulation phase, while relevant metabolic engineering can increase PHB production yield and broaden the applicable carbon sources. Furthermore, more engineering efforts of technology scale-up need to be placed to improve the industrial potential of PHB production.
